# Atrial Septal Defect (ASD) Repair Unveiling an Unusual Conduction Conundrum: A Wenckebach Case Report

**DOI:** 10.7759/cureus.62073

**Published:** 2024-06-10

**Authors:** Matthew J Van Ligten, Douglas E Rappaport, Lauren B Querin, Wayne A Martini

**Affiliations:** 1 Emergency Medicine, Mayo Clinic Alix School of Medicine, Scottsdale, USA; 2 Emergency Medicine, Mayo Clinic Arizona, Phoenix, USA

**Keywords:** cardiology, stroke risk, arrhythmia, eisenmenger syndrome, congenital heart disease, av block, pulmonary hypertension, mobitz type 1 wenckebach, percutaneous intervention, atrial septal defect (asd)

## Abstract

Atrial septal defects are a common congenital malformation that can lead to an elevated risk for stroke due to the bypass of the lungs by deep vein thrombosis, as well as the expected repercussions of pulmonary hypertension if left untreated. Surgical intervention is definitive; however, recent advancements in treatment options, such as percutaneous intervention, represent a safer and equally effective way to treat this congenital complication. While safer, percutaneous interventions can also lead to adverse events that may force patients to present to the emergency department. Here, we present a unique case of a patient with congenital atrial septal defect status post-percutaneous intervention who developed a new-onset second-degree AV block, Mobitz type 1 Wenckebach rhythm.

## Introduction

Atrial septal defects (ASDs) are among the most common congenital heart defects, characterized by an opening in the atrial septum that allows blood to flow between the left and right atria. This anomaly can lead to significant clinical complications, including pulmonary hypertension and an increased risk of stroke due to paradoxical embolism [1]. Traditional surgical repair has long been the definitive treatment; however, advancements in percutaneous intervention have introduced safer, minimally invasive alternatives that are equally effective [2,3]. Despite these advancements, percutaneous interventions are not without risks, and patients may experience adverse events post-procedure. This case report details the presentation, management, and resolution of a unique complication following percutaneous ASD closure: a new-onset second-degree AV block, Mobitz type 1 Wenckebach rhythm. The report aims to highlight the importance of vigilant monitoring and follow-up in managing such complications to ensure optimal patient outcomes.

## Case presentation

A 42-year-old female with a history of recently repaired congenital ASD presented with a chief complaint of intermittent palpitations for the last two hours. She reported shortness of breath with walking and had noticed unusual heart patterns on her smart watch. She denied any history of previous arrhythmias. Two days prior to coming into the ED, the patient had undergone a percutaneous secundum ASD closure secondary to elevated right heart pressures thought to be due to a left-to-right intracardiac shunt and to prevent stroke risk in the future. Prior to the intervention, the patient was experiencing decreased exercise tolerance due to her pulmonary hypertension. She recovered well following the procedure. 

Her preoperative echocardiography revealed normal left atrial size, with no evidence of a left atrial appendage thrombus. The right atrium was found to be enlarged, and there was a moderate secundum atrial septal defect with dimensions of 1.68 cm x 0.60 cm in diastole and 1.5 cm x 0.87 cm in systole, accompanied by a continuous left-to-right shunt. The left ventricle had a normal chamber size with an estimated ejection fraction of 55%, and no regional wall motion abnormalities were noted (Video 1).

**Video 1:** Preoperative echocardiogram showing ASD ASD: atrial septal defect

**Figure 1:**
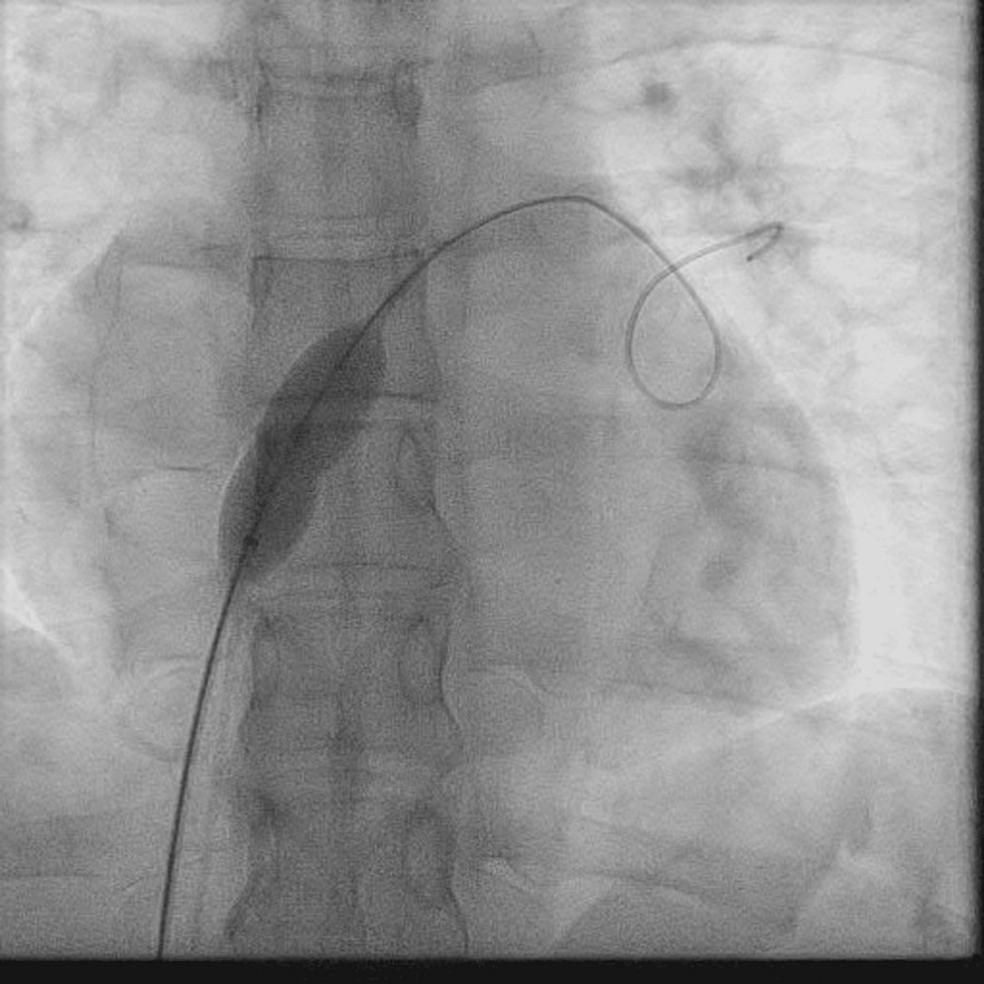
ASD identification with closure placement device ASD: atrial septal defect

**Figure 2:**
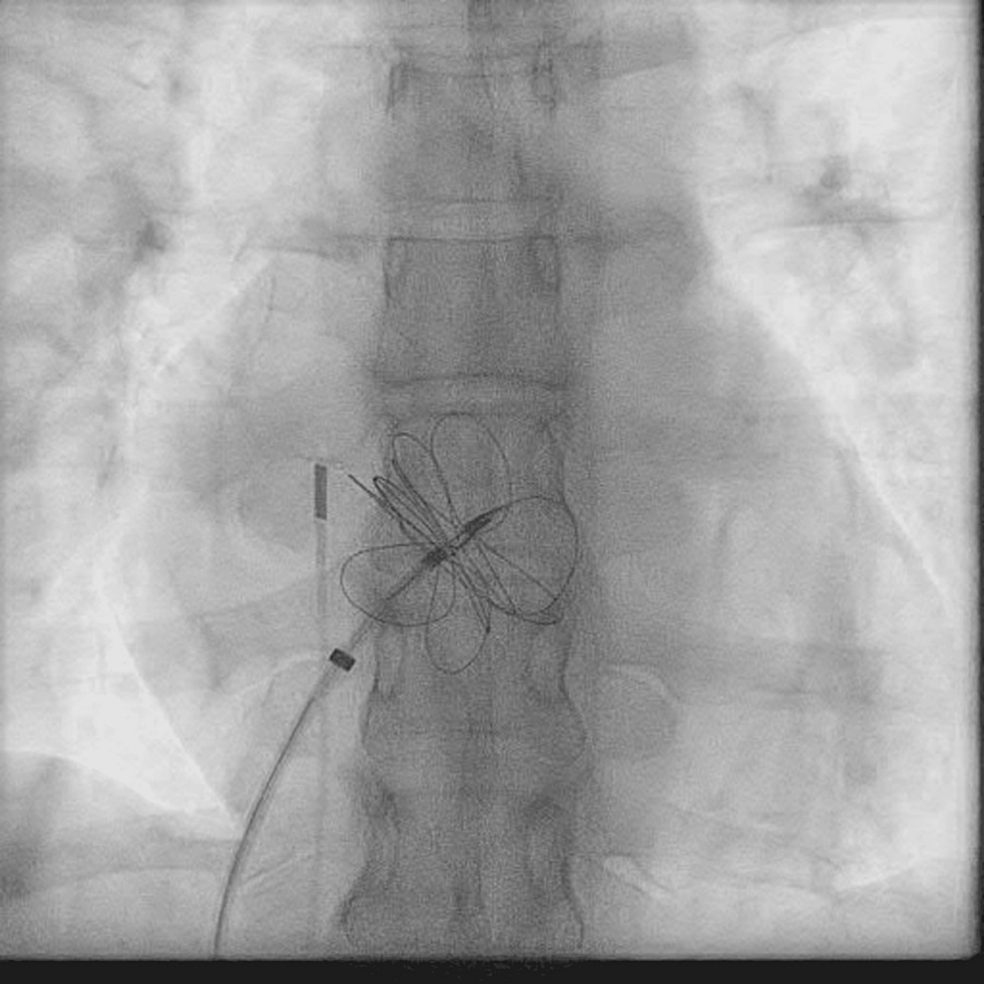
Expansion of ASD occluder during implantation ASD: atrial septal defect

**Figure 3:**
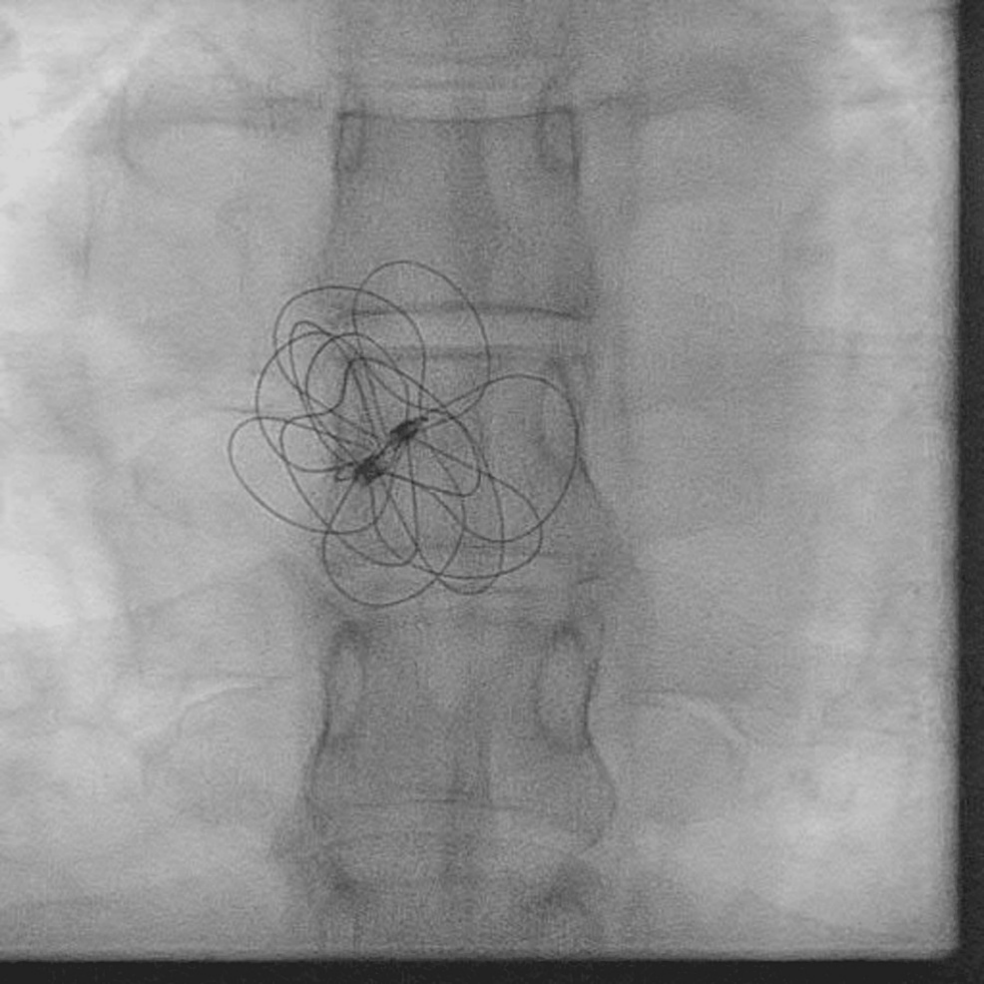
ASD occluder device securely in place ASD: atrial septal defect

The patient received a transcutaneous ASD occluder device with a GORE CARDIOFORM ASD Occluder of 37 mm. Perioperative fluoroscopy was used to identify the ASD site (Figure 1) as well as the implantation (Figure 2) of the device, with visualization of the device in a stable position (Figure 3). Postoperative echocardiography revealed the ASD occlusion device in a stable position. No residual color flow across the interatrial septum was noted (Video 2).

**Video 2:** Post-operative echocardiogram showing ASD closure ASD: atrial septal defect

**Figure 4:**
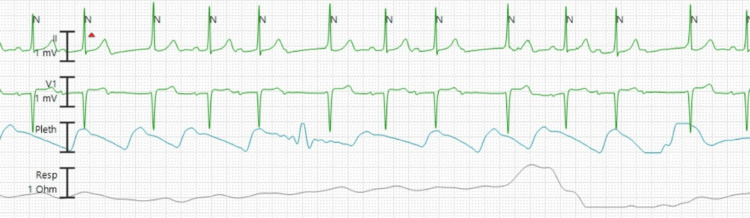
Telemetry reading showing second-degree heart block, Mobitz type 1 (Wenckebach)

**Figure 5:**
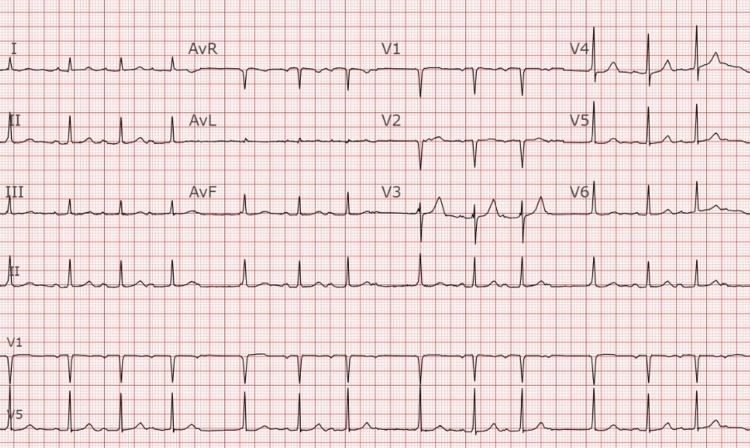
ECG showing second degree heart block, Mobitz type 1 (Wenckebach)

Upon arrival at the ED, her vitals were stable, with a normal rate of 92 beats per minute, an oxygen saturation of 100% on room air, and a blood pressure of 110/71 mmHg. The physical exam was notable for an irregular heartbeat, and subsequent telemetry (Figure 4) and electrocardiogram (Figure 5) demonstrated a Mobitz type 1 block, Wenckebach. Further imaging via chest x-ray showed no signs of pneumonia with a correctly positioned ASD closure device. Laboratory workup was notable for a stable hemoglobin (14.9 g/dL), a slight elevation of the white blood cell count at 11.0 x 10(9)/L (3.4-9.6), and a thyroid stimulating hormone level within normal limits at 1.31 mIU/L (0.30-4.20). N-terminal pro-B-type natriuretic peptide (NT pro-BNP) was elevated for her age at 839 pg/mL (<162). Initial and repeat troponin were found to be stable at 8 ng/L (<10). An echocardiogram done at the bedside demonstrated borderline enlarged RV size and a stable ASD device that had no residual flow across the defect.

Due to the patient's recent percutaneous ASD closure and history of congenital heart disease, cardiology was consulted. They recommended outpatient follow-up with a one-week heart monitor, as it is common to see second-degree AV blocks with Mobitz type II, which resolve within three to six months following cardiac interventions. They felt as though the patient’s symptoms and her Mobitz-1 were in a stable rhythm and that observation would be an appropriate plan. Outpatient follow-up revealed spontaneous resolution of the arrythmia by one month after her procedure.

## Discussion

Atrial septal defects (ASDs) rank as the second most common congenital heart defect, affecting three-nine per 10,000 live births and comprising 6-10% of congenital cardiac defects [4,5]. At birth, patients are typically asymptomatic due to left-to-right shunting within the cardiac chambers. Despite this initial lack of symptoms, timely treatment of ASDs is crucial, as 60% of these defects will increase in size as the patient ages. This enlargement leads to enhanced left-to-right shunting, which can result in pulmonary hypertension by the fourth or fifth decade of life. [4] Among those who develop pulmonary hypertension, approximately 10% may progress to Eisenmenger syndrome, where the shunt flow reverses due to significant pulmonary hypertension, causing cyanosis and heart failure symptoms. Increased pressures in the right atrium or ventricle often necessitate surgical intervention to prevent shunt reversal and the development of Eisenmenger syndrome.

Definitive treatment for ASDs can be achieved via percutaneous or surgical intervention. Percutaneous procedures have shown effectiveness comparable to surgery but with lower complication rates, making them a safer option for many patients. [2] The success of these interventions is exemplified by cases where advanced imaging techniques like MRI and transesophageal echocardiogram (TEE) facilitate accurate diagnosis and guide minimally invasive procedures, ensuring optimal outcomes and minimizing complications. Additionally, patients with ASDs have a significantly elevated risk of stroke, with an incidence of approximately 10% for those without concomitant arrhythmias. [6]

While procedural or surgical intervention is the mainstay of ASD care, patients who choose to forgo these options can mitigate stroke risk through oral anticoagulation and rhythm control, typically using beta blockers if symptomatic with an atrial arrhythmia. These patients should undergo repeat echocardiograms every three years to monitor for elevated right heart pressure. Significant elevations in right atrial or ventricular pressures often lead to surgical intervention to prevent shunt reversal and Eisenmenger syndrome. [5] Research indicates that post-surgical intervention, 8% of patients will experience arrhythmias, with 3% developing atrial fibrillation or atrial flutter and 6% experiencing palpitations. [7] While second-degree atrioventricular (AV) block is a rare surgical complication, it is typically self-resolving within three months of the procedure. [8]

The management of ASD also involves addressing its potential complications. Advanced imaging, including, but not limited to, cardiac computed tomography, assists in precise device sizing, helping prevent severe complications like hemopericardium. Combination therapies, incorporating PAH-specific medications followed by surgical ASD closure, exemplify a comprehensive approach to managing complex cases with concurrent pulmonary arterial hypertension [9].

## Conclusions

Atrial septal defects represent a common congenital cardiac anomaly that is often asymptomatic at birth. If left untreated, the patient can develop pulmonary hypertension, leading to shunt reversal and cyanosis, and carry a significantly higher risk of thromboembolic stroke. Definitive management includes surgical replacement or percutaneous intervention associated with symptom improvement and adverse effects, such as arrhythmia. These arrhythmias can lead patients to present to the emergency department and require a different treatment paradigm compared to classic teaching with close follow-up with the cardiology team.
